# A homozygous missense variant in the YG box domain in an individual with severe spinal muscular atrophy: a case report and variant characterization

**DOI:** 10.3389/fncel.2023.1259380

**Published:** 2023-09-26

**Authors:** Leping Li, Lalith Perera, Sonia A. Varghese, Yael Shiloh-Malawsky, Senyene E. Hunter, Tam P. Sneddon, Cynthia M. Powell, A. Gregory Matera, Zheng Fan

**Affiliations:** ^1^Biostatistics and Computational Biology Branch, National Institute of Environmental Health Sciences, Durham, NC, United States; ^2^Genome Integrity and Structural Biology Laboratory, National Institute of Environmental Health Sciences, Durham, NC, United States; ^3^Division of Pediatric Neurology, Department of Neurology, University of North Carolina at Chapel Hill, Chapel Hill, NC, United States; ^4^Department of Pathology and Lab Medicine, University of North Carolina at Chapel Hill, Chapel Hill, NC, United States; ^5^Division of Genetics and Metabolism, Department of Pediatrics, University of North Carolina at Chapel Hill, Chapel Hill, NC, United States; ^6^Integrative Program for Biological and Genome Sciences, University of North Carolina at Chapel Hill, Chapel Hill, NC, United States

**Keywords:** spinal muscular atrophy (SMA), c.796T>C variant, g.27134T>G polymorphism, African American, non-deletion, modeling, YG Box

## Abstract

The vast majority of severe (Type 0) spinal muscular atrophy (SMA) cases are caused by homozygous deletions of survival motor neuron 1 (*SMN1*). We report a case in which the patient has two copies of *SMN1* but clinically presents as Type 0 SMA. The patient is an African American male carrying a homozygous maternally inherited missense variant (c.796T>C) in a *cis*-oriented *SMN1* duplication on one chromosome and an *SMN1* deletion on the other chromosome (genotype: 2*+0). Initial extensive genetic workups including exome sequencing were negative. Deletion analysis used in the initial testing for SMA also failed to detect SMA as the patient has two copies of *SMN1*. Because of high clinical suspicion, SMA diagnosis was finally confirmed based on full-length *SMN1* sequencing. The patient was initially treated with risdiplam and later gene therapy with onasemnogene abeparvovec at 5 months without complications. The patient’s muscular weakness has stabilized with mild improvement. The patient is now 28 months old and remains stable and diffusely weak, with stable respiratory ventilatory support. This case highlights challenges in the diagnosis of SMA with a non-deletion genotype and provides a clinical example demonstrating that disruption of functional SMN protein polymerization through an amino acid change in the YG-box domain represents a little known but important pathogenic mechanism for SMA. Clinicians need to be mindful about the limitations of the current diagnostic approach for SMA in detecting non-deletion genotypes.

## Introduction

Spinal muscular atrophy (SMA) is a rare autosomal recessive disorder that results from loss of specialized nerve cells in the brainstem and spinal cord (motor neurons) (Prior et al., 1993; Kolb and Kissel, 2015; Mercuri et al., 2022). The pathogenic mechanism of SMA involves dysfunction of motor neurons located in the anterior horn cells of the spinal cord and brain stem nuclei. Motor neurons are responsible for receiving neuronal inputs from the brain and transmitting the information via the spinal cord to the peripheral nerves innervating the muscles. Disruption, or lack of transmission, of spinal nerve impulses to the peripheral system leads to weakness and ultimately atrophy of multiple muscle groups that are essential for ambulation, respiration, and swallowing (Lefebvre et al., 1997). Left untreated, these deficits result in increased mortality and morbidity (Kolb and Kissel, 2015).

The spectrum of SMA disease severity ranges from Type 0, prenatal onset with weakness and respiratory failure at birth, and Type 1 infantile onset with onset in the first 6 months of life, to Type 4 late-onset with milder phenotypes and a slow rate of progression (Mercuri et al., 2022). Recently developed disease modifying treatments include gene therapy and therapies to increase SMN protein production. These treatments are used along with treatments targeting symptom management and prevention of complications (Talbot and Tizzano, 2017; Jablonka et al., 2022).

In most cases, SMA is caused by homozygous deletion of the survival motor neuron 1 (*SMN1*) gene (Lefebvre et al., 1995; Alias et al., 2009). In a small number of cases, SMA can be caused by point mutations in *SMN1* (Prior, 2007; Wirth, 2021). The severity of SMA depends on the availability of functional SMN protein; those with the lowest levels of functional SMN protein display the most severe phenotype (Prior et al., 1993).

In this communication, we report homozygosity for a rare missense variant in *SMN1* in an individual with prenatal onset SMA (Type 0). We describe the complex genotypes of the parents and the affected proband. We also provide *in silico* predictions for the pathogenic effect of the variant. Finally, we highlight the challenges to diagnose such individuals and the limitations of current SMA diagnostics (Ben-Shachar et al., 2011) for individuals with non-deletion genotypes. Our experience should help other clinicians in a similar situation.

## Case presentation and initial assessments

An African American male infant was born at gestational age 38 weeks and 4 days with severe hypotonia, weakness, poor feeding, and respiratory failure that required intubation and ventilatory support at the age of 3 weeks at an outside hospital. Extensive genetic workups obtained at the outside hospital were negative, included a DNA methylation study for Prader Willi/Angelman syndromes, chromosomal microarray analysis, and exome sequencing. Genetic testing for SMA indicated that the patient had two copies of *SMN1*.

The patient was transferred to our medical center at the age of 3 months for further evaluation. Upon arrival, the patient was noted to be non-dysmorphic with normal head circumference, with an ~80th percentile length and ~20th percentile weight. On examination, the infant was visually alert, able to recognize his parents, and presented social smiles. The patient had severe hypotonia and muscular weakness with minimal spontaneous movements. The patient had absent deep tendon reflexes throughout, but responded to touch on the face and limbs. Due to significant respiratory failure and feeding difficulty, tracheostomy G-tube placement and a biopsy of the thigh muscle were carried out at 3 months and 1 week of age.

## Further diagnostic assessment

Muscle biopsy of vastus lateralis showed “probable denervation atrophy.” Comments added to the interpretation included “The pattern of large fibers being Type 1 and the atrophic fibers of either histochemical fiber type can be seen in association with SMA.”

Brain magnetic resonance imaging and metabolic screenings were carried out; both were normal. However, NCS/EMG (nerve conducting studies/electromyography) showed severe sensory and motor polyneuropathy. Motor evoked responses in the left fibular nerve were absent, recorded at the extensor digitorum brevis (EBD) and tibialis anterior (TA), and the left tibial, median and ulnar nerves. Motor evoked responses were absent in the left facial nerve recorded at the nasalis, orbicularis rois and oculi muscles. Sensory evoked responses were absent in the left median, ulnar, radial, sural and superficial fibular nerves. Needle EMG of selected muscles in the left arm and leg showed active denervation in all tested muscles. No motor unit potential (MUP) activation was noted in the first dorsal interosseous (FDI) muscle.

The NCS/EMG pattern of motor and sensory neuropathy can also be seen in severe SMA. The patient had prominent respiratory failure that is rare in hereditary polyneuropathy. These findings suggest that the patient does not have congenital Charcot–Marie–Tooth (CMT) for which facial nerve involvement is rare and most affected nerves are distal.

Clinically, the patient continued to show a decline in respiratory function that required more ventilator support during night and day. Due to the clinical presentation of Type 0 SMA and the subsequent support from muscle biopsy findings, genetic workups were expanded. A reanalysis of whole exome sequencing (WES) study was submitted with a focus on genes related to SMA. In addition, hereditary neuropathy analysis was requested. Results for both analyses were negative. Sequencing study for the *SMARD* (*IGHMBP2*) gene also came back negative. Additional tests including trinucleotide repeat expansion tests for myotonic dystrophy Type I and deletion/duplication and sequencing tests for mitochondrial genome were normal.

**Figure 1:**
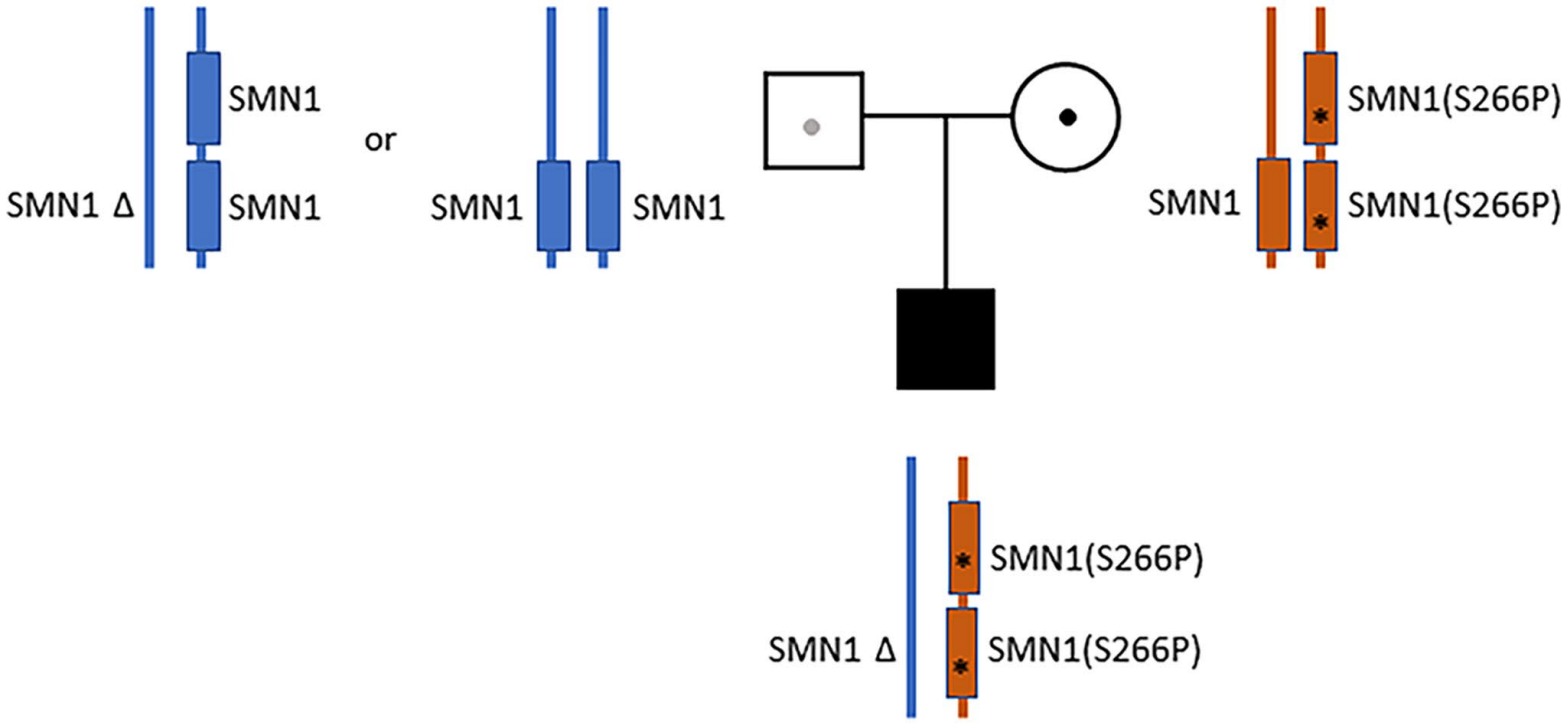
Parental and proband *SMN1* genotypes. The father (blue) has two copies of *SMN1* without the c.796T>C variant or the g.27134T>G polymorphism (1+1 or 2+0). The mother (orange) has a *cis*-oriented *SMN1* duplication with the homozygous c.796T>C variant (marked by “*”) on one allele and a wild-type *SMN1* on the other allele (2*+1). The proband has a *cis*-oriented *SMN1* duplication with the homozygous c.796T>C variant on one allele and a null *SMN1* on the other allele (2*+0). The proband’s null *SMN1* on the paternal chromosome was presumed to have arisen from a *de novo* deletion. Alternatively, the father may also be a 2+0 carrier, but is without the g.27134T>G polymorphism.

Despite the initial negative SMA tests from the outside hospital, clinical presentations continued to suggest motor neuron disease. Additional sequence analysis including deletion and duplication of *SMN1* and *SMN2* were ordered. The test showed that the infant has one copy of *SMN2*. A variant of unknown zygosity, c.796T>C (p.Ser266Pro) present on either *SMN1* or *SMN2*, was identified and classified as a variant of uncertain significance. Given the uncertainty, full-length Sanger sequencing analysis of *SMN1* was ordered. The sequencing results were complex, and the report was revised by the lab three times. In the final report, a homozygous c.796T>C variant on *SMN1* (Prior, 2007) was identified and classified as “likely pathogenic.” Notably, the g.27134T>G polymorphism (Luo et al., 2014) was also identified in the patient. Subjects with this genotype are often referred to as “silent” carriers of SMA—as they carry two copies of *SMN1* on the same haplotype (Luo et al., 2014). Collectively, the results suggested that the patient carries a *cis* duplication of *SMN1* on one chromosome and a null *SMN1* allele on the other chromosome (2*+0) (Sugarman et al., 2012; Feng et al., 2017). Parental analysis of the mother revealed a complex genotype (2*+1)—with three copies of *SMN1,* heterozygous for the c.796T>C variant, and also carrying the g.27134T>G polymorphism (Figure 1). A detailed depiction of the parental and proband genotypes is provided in Supplementary Figure S1. The mother also has one copy of *SMN2*. Paternal analysis showed that the father has two copies of *SMN1* without the c.796T>C variant or the g.27134T>G polymorphism. The father also has only one copy of *SMN2* (Figure 1; Supplementary Figure S1). These findings suggest that the proband’s homozygous duplication of the *SMN1* variant (c.796T>C), along with the g.27134T>G polymorphism, were inherited maternally. The proband’s haploinsufficiency of *SMN1* on the paternal chromosome was presumed to have arisen from a *de novo* deletion of the copy of *SMN1* from the paternal homolog. Alternatively, the father may also be a 2+0 carrier, but is without the g.27134T>G polymorphism (Figure 1; Supplementary Figure S1).

**Figure 2:**
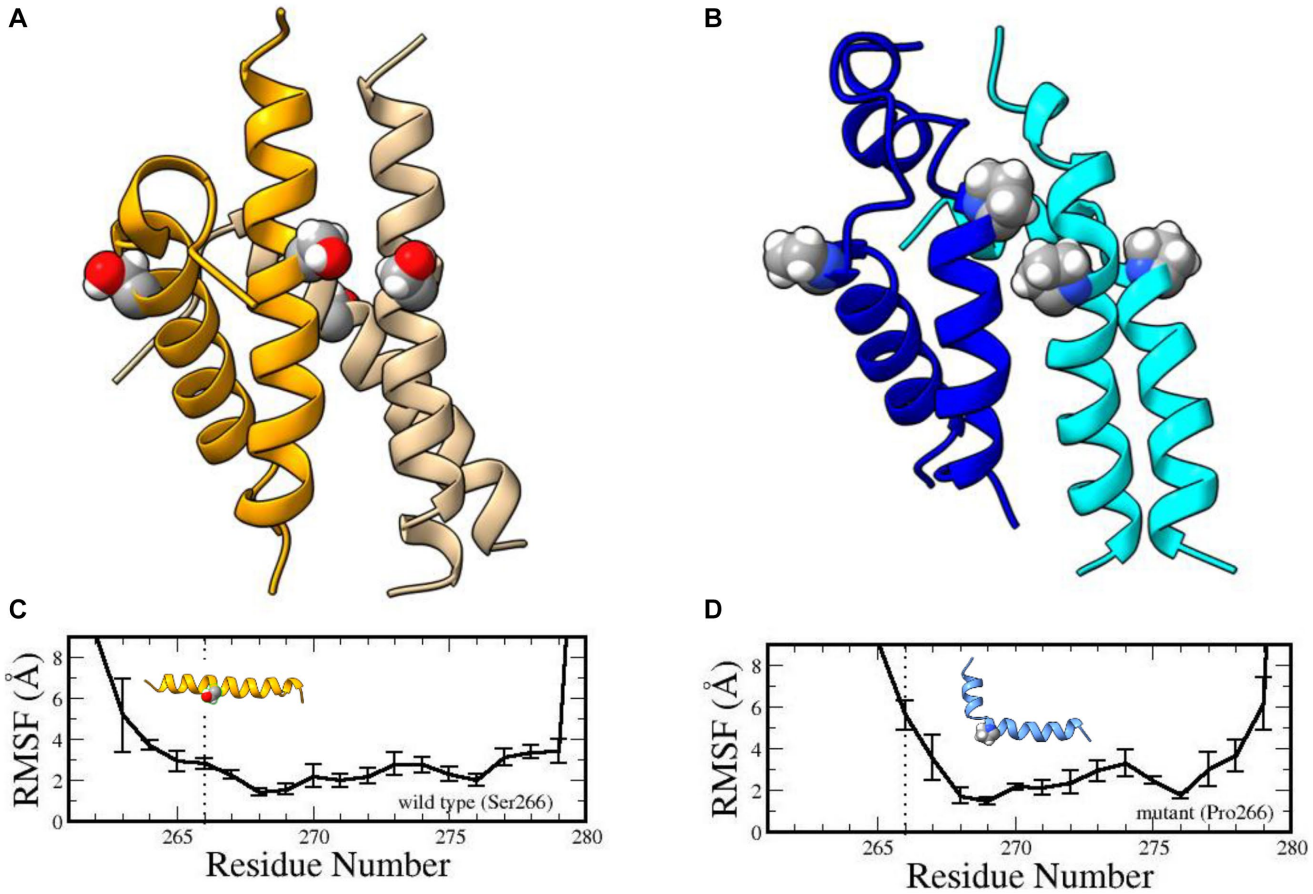
Structures of human wild-type and mutant SMN YG-box (residues 255–281) tetramers. Ser266 in the wild-type peptide and Pro266 in the mutant peptide are shown in ball-and-stick representations. Predicted solution structures of the wild-type YG-box tetramer (**A**, golden/wheat) and the mutant tetramer (**B**, blue/cyan). Root mean squared fluctuations of the helix backbones in the wild-type YG-box (**C**) and the mutant YG-box (**D**) from molecular dynamics simulations. The vertical dotted lines indicate the location of the substitution. The error bars indicate standard deviations. A kink is noticeable in the mutant peptide but not in the wild-type peptide.

Phylogenetically speaking, the YG box is the most highly conserved subdomain of SMN and performs an essential function in self-oligomerization (Lorson et al., 1998; Gupta et al., 2021). The human YG box dimer adopts an alpha-helical structure (Martin et al., 2012) that does not directly involve Ser266. Instead, this residue forms part of a motif that is required for higher-order multimerization (Gupta et al., 2021). The so-called small- or s-motif includes Ser266 and Ser270 and, *in vivo*, these residues do not tolerate large, bulky substitution mutations such as Ser266Gln or Ser270Phe [Gupta et al. (2021) and references therein]. To probe the effect of the variant on YG-box tetramerization, we carried out extensive molecular dynamics simulations (Supplementary material) on both the wild-type and mutant YG-box peptides. Compared to its wild-type counterpart, our molecular dynamics simulation studies suggest that this mutation is energetically unfavored to form oligomers. Microsecond dynamics simulations indicated that the Ser266Pro YG-box helix structure becomes disordered, especially in the tetramer complex (Figure 2). Furthermore, proline is a known helix breaker, and a kink in the YG-box at position 266 is predicted to profoundly alter the wild-type SMN structure (Figure 2). These results suggest that both the dimer and tetramer complexes of the Ser266Pro mutant YG-box would be much less stable than those of its wild-type counterpart.

## Treatments

Although the c.796T>C variant was considered to be of unknown significance by the initial lab tests, there remained a high degree of clinical suspicion for SMA. Due to the severe clinical presentation of neuromuscular disease, the patient was treated with oral risdiplam for SMA at our medical center while the additional genotyping tests were being conducted. Prior to gene therapy, motor outcome measures were obtained. The patient scored 1/26 for Hammersmith Infant Neurological Examination (HINE) and 10/64 for The Children’s Hospital of Philadelphia Infant Test of Neuromuscular Disorders (CHOP INTEND). Gene therapy with onasemnogene abeparvovec was initiated when it was clear that the patient carried only a single copy of *SMN2*, and that the *SMN1* c.796T>C variant was homozygous and likely pathogenic. At 5 months old, the patient received gene therapy without complications. The patient’s muscular weakness has stabilized with mild improvement. The patient was discharged home at the age of five and a half months and has been managed by local health providers since he lives in a remote area many hours from our hospitals. Because of this, his outcome measures are not monitored by our hospitals. The family has kept in communication with us. The patient exhibited improvement of respiratory function (required less vent support) after the gene therapy treatment. The patient is now 28 months old and remains stable, diffusely weak, receiving full gastrostomy tube feeds and respiratory ventilatory support that requires less pressure support compared to the time at discharge. The child’s weight has improved, and his cognitive function appears to remain intact. The patient is apparently doing well; his parents tell us he has enjoyed social outings, pool time, and visits to the zoo this summer.

**Figure 3:**
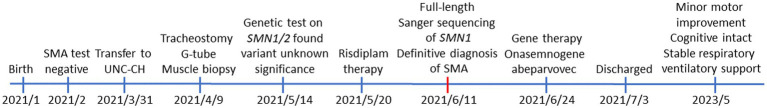
The timeline of clinical presentations, diagnosis, and treatment for the patient from birth to current age (28 months old).

The timeline of the patient from birth—including clinical presentations, initial genetic testing, final diagnosis, treatments, and the current status—is provided in Figure 3.

## Discussion

Certain aspects of the clinical presentation, especially the electrophysiological findings, might point to the patient having sensory and motor neuropathy. It has been suggested that Type I SMA can have electrophysiology findings mimicking mixed neuropathy (Rudnik-Schöneborn et al., 2003; Anagnostou et al., 2005; Fernández-Torre et al., 2008). On the other hand, younger Type I SMA patients have been shown to have normal sensory nerve conduction velocities and sensory nerve action potential (SNAP) amplitudes in the sural and median nerves (Pro et al., 2021). It is also worth noting that our patient has the most severe form of SMA, Type 0. To our knowledge, no studies have reported on sensory electrophysiological findings specific to SMA Type 0. Extensive genetic workouts confirmed that the patient carries a homozygous mutation in the critical YG-box domain, rendering the SMN protein non-functional. Prior to receiving SMA treatments, the patient had progressive clinical decline in muscular strength as well as worsening respiratory status and issues gaining weight despite tube feeding. It is clinically significant that the patient has stabilized, requires less ventilator support, and gained healthy weight after ridisiplam treatment and later the initiation of gene therapy. The patient continues to be clinically stable.

The vast majority of severe SMA cases are caused by homozygous deletions of *SMN1* (Prior et al., 1993; Alias et al., 2009; Keinath et al., 2021; Wirth, 2021; Li et al., 2022; Mercuri et al., 2022). We report a case in which the patient carries a homozygous missense variant (c.796T>C) on *SMN1* that clinically presents as Type 0 SMA. The Ser266Pro variant on *SMN1* is not new (ClinVar accession: VCV000634939.2) (Prior, 2007). However, the original report provided no additional information (i.e., the *SMN1* and *SMN2* copy numbers, the clinical presentation in SMA, the carrier haplotypes, and the effect of the variant on SMN protein self-oligomerization).

For the individual reported here, parental testing showed that the mother has 3 copies of *SMN1*, and is heterozygous for the c.796T>C variant. The mother also has the g.27134T>G polymorphism (Luo et al., 2014), indicative of an *SMN1* duplication on one chromosome and one wild-type *SMN1* on the other chromosome (2*+1) (Sugarman et al., 2012; Feng et al., 2017). The father has 2 copies of *SMN1* and does not carry the g.27134T>G polymorphism (1+1). Taken together, the genetic data suggest that the proband inherited the 2*+0 genotype with the homozygous c.796T>C variant from the mother. Although the father has two copies of *SMN1*, his *SMN1* haplotype is uncertain. The father could have either the 1+1 (one copy of *SMN1* on each chromosome) or the 2+0 genotype, and lacking the g.27134T>G polymorphism.

The pathogenic variant, c.796T>C (p.Ser266Pro), is located in the highly conserved YG-box domain important for SMN self-oligomerization (Lorson et al., 1998; Gupta et al., 2021). Genetic manipulations of amino acids in this domain have established a direct correlation between oligomerization and SMA clinical type (Lorson et al., 1998). Substituting Ser130 (human ortholog Ser266) to Gln in fission yeast abolished SMN protein tetramerization (Gupta et al., 2021).

When multiple copies of *SMN2* are present, therapeutic agents such as nusinersen and risdiplam can be effective by increasing functional SMN protein through correcting *SMN2* exon 7 splicing (Talbot and Tizzano, 2017; Groen et al., 2018; Jablonka et al., 2022; Mercuri et al., 2022). Such therapy is less effective, however, for patients with just one copy of *SMN2*. Gene therapy should be more effective, this is the case for our patient. Onasemnogene abeparvovec was infused at age of 5 months without complications. At age of 28 months, the patient’s condition remains stable with minor improvements in motor skills and respiratory status, despite being Type 0. This patient appears to remain cognitively intact. We believe this may be the first published report of an infant with SMA Type 0 who has been treated with onasemnogene abeparvovec, and the first report of SMA Type 0 treated with risdiplam.

The genotypes of the African American family reported herein are complex, making SMA diagnosis challenging. African American populations are known to display a higher prevalence of the silent 2+0 carrier genotype compared to other ethnic groups (Sugarman et al., 2012; Feng et al., 2017). The 2+0 genotype can give clinicians the false impression that two functional copies of *SMN1* genes are present, and thus, they are less likely to suspect SMA. This complication may, in part, explain why the SMA detection frequency for African Americans (~71%) was lower than that for non-black population (87%–95%) (MacDonald et al., 2014).

Finally, our case study reiterates the limitations of the current molecular diagnostic approaches for SMA with a non-deletion genotype (Ben-Shachar et al., 2011). Deletion analysis used in the initial testing for SMA (i.e., newborn screening), is beneficial but it does not detect point mutations. When clinical suspicion for SMA is high, we recommend full-length Sanger sequencing for *SMN1*. Early diagnosis of and disease modifying intervention for SMA can save lives, as effective therapies are available (Farrar et al., 2017; Talbot and Tizzano, 2017; Coratti et al., 2021; Jablonka et al., 2022).

This study is limited by the lack of functional biochemical assay of the mutant SMN protein. The missense variant (c.796T>C) is located in the YG-box domain where other variants in the region have been shown to disrupt SMA oligomerization (Lorson et al., 1998; Gupta et al., 2021). Although we have provided *in silica* evidence for the variant, further functional assay using expressed mutate protein would be needed to mechanistically confirm the effect of the variant on SMA. Notably, however, mutations at this aa residue, e.g., to a Gln, have been analyzed in model organisms and the phenotype is consistent (Gupta et al., 2021).

## Conclusion

Most severe cases of SMA are caused by homozygous deletion of *SMN1*; only 2% to 5% of cases are due to heterozygous *SMN1* deletion on one allele and a point mutation in *SMN1* on the other allele (Prior et al., 1993; Alias et al., 2009; Keinath et al., 2021; Wirth, 2021; Mercuri et al., 2022). Here, we report the first case of SMA with a homozygous *SMN1* duplication expressing a single missense variant, c.796T>C (p.Ser266Pro). We present *in silico* evidence suggesting that the pathogenic variant prevents SMN proteins from forming stable functional oligomers.

Our case demonstrates the limitations of and challenges for current diagnostic screening tests for SMA in non-deletion genotypes (MacDonald et al., 2014). The African American population has a relatively high prevalence of the 2+0 genotype (Sugarman et al., 2012; Feng et al., 2017). *SMN1* deletion analysis alone can miss SMA diagnosis. Full gene sequencing of *SMN1* should be considered for the 2+0 genotype, especially when the clinical suspicion for SMA is high. Accurate molecular diagnosis has implications for timely and appropriate treatment of the disease.

## Data availability statement

The raw data supporting the conclusions of this article will be made available by the authors, without undue reservation.

## Ethics statement

Written informed consent was obtained from the individual(s) for the publication of any potentially identifiable images or data included in this article.

## Author contributions

LL: Formal analysis, Methodology, Writing – original draft. LP: Formal analysis, Investigation, Methodology, Writing – review & editing. SV: Investigation, Writing – original draft. YS-M: Investigation, Writing – review & editing. SH: Investigation, Writing – review & editing. TS: Investigation, Writing – review & editing. CP: Investigation, Writing – review & editing. AM: Formal analysis, Investigation, Writing – review & editing. ZF: Conceptualization, Formal analysis, Investigation, Project administration, Writing – original draft, Writing – review & editing.

## Funding

The author(s) declare financial support was received for the research, authorship, and/or publication of this article. This research was supported in part by the Intramural Research Program of the National Institutes of Health, National Institute of Environmental Health Sciences (ZIA ES101765 to LL and ZIA ES43010 to LP) and by the National Institute of General Medical Sciences (R35-GM136435 to AM).

## Conflict of interest

The authors declare that the research was conducted in the absence of any commercial or financial relationships that could be construed as a potential conflict of interest.

The author(s) declared that they were an editorial board member of Frontiers, at the time of submission. This had no impact on the peer review process and the final decision.

## Publisher’s note

All claims expressed in this article are solely those of the authors and do not necessarily represent those of their affiliated organizations, or those of the publisher, the editors and the reviewers. Any product that may be evaluated in this article, or claim that may be made by its manufacturer, is not guaranteed or endorsed by the publisher.
