# Supplementary material for: A homozygous missense variant in the YG box domain in an individual with severe spinal muscular atrophy: a case report and variant characterization
Source: Front Cell Neurosci. 2023 Sep 26;17:1259380. doi: 10.3389/fncel.2023.1259380 (PMC10571918; doi:10.3389/fncel.2023.1259380)
Supplement: Supplementary file 1 [file Data_Sheet_1.docx]

Supplementary materials for

**Identification and *in silico* Characterization of a Homozygous Missense Variant in the YG box Domain in an Individual with Severe Spinal Muscular Atrophy**

Leping Li, Lalith Perera, Sonia A. Varghese, Yael Shiloh-Malawsky, Senyene E. Hunter, Tam P. Sneddon, Cynthia M. Powell, A. Gregory Matera, Zheng (Jane) Fan


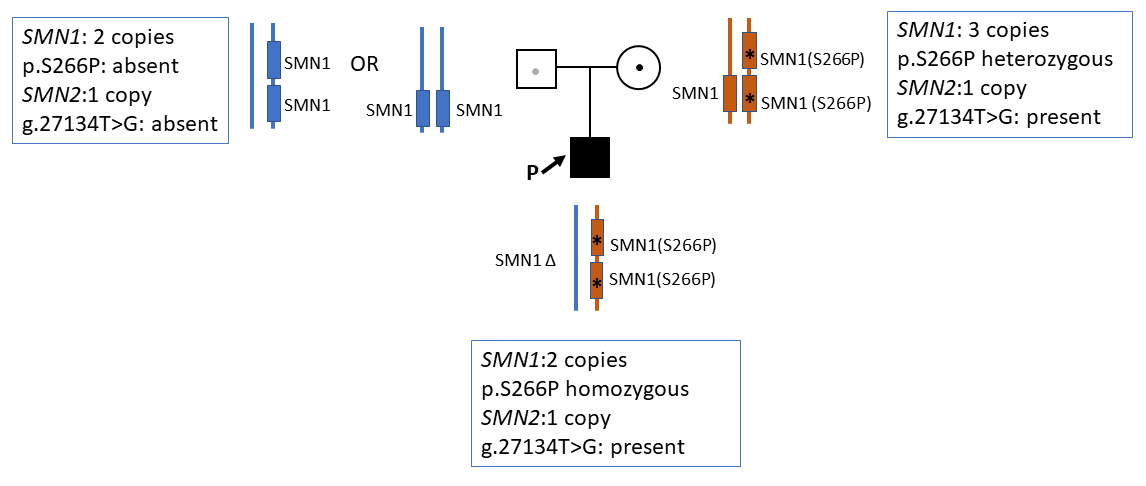


**Figure S1**. Depiction of the parental and proband genotypes. The person’s father (blue) has two copies of *SMN1* without the c.796T>C variant or the g.27134T>G polymorphism (1+1 or 2+0). The person’s mother (orange) has three copies of *SMN1* with the homozygous c.796T>C variant (marked by ‘*’) on one allele and a wild-type *SMN1* on the other allele (2*+1). The proband has a *cis*-oriented *SMN1* duplication with the homozygous c.796T>C variant on one allele and a null *SMN1* on the other allele (2*+0). The proband’s null *SMN1* on the paternal chromosome was presumed to have arisen from a *de novo* deletion of *SMN1*. Alternatively, the father may also be a 2+0 carrier, but lacks the g.27134T>G polymorphism.

**MATERIALS AND METHODS**

**Sequence analysis**

Initial sequence analysis and deletion/duplication testing on *SMN1/2* (Invitae Spinal Muscular Atrophy Panel) were carried out by Invitae Inc. (San Francisco, CA). Full-length Sanger sequencing of *SMN1* was carried out by Mayo Clinic Laboratories (Rochester, MN).

**Molecular modeling and dynamics simulations**

The starting structure of the human SMN YG-box dimer (residues 255-281) was based on the crystal structure of the human SMN YG-dimer (PDB ID: 4GLI, Resolution: 1.90 Å) (<https://doi.org/10.2210/pdb4GLI/pdb>). Missing atoms and protons were introduced using the leap module of the Amber2020 software ^1^. The Ser266Pro substitution was introduced using Modeller-10.2 ^2^. As described in the work of Gupta et al ^3^, we used the helix tetramer configuration of the crystal structure of the *E. coli* glycerol facilitator protein (PDB ID: 1FX8) (<https://doi.org/10.2210/pdb1FX8/pdb>) as the template for the SMN YG-box tetramer.

Twenty-six Na^+^ and Cl^−^ ions were added to provide the 100mM effective ionic concentration of the simulation systems. An additional four Na^+^ ions were introduced to neutralize the charges of the peptides.

Both the wild-type and mutant YG-box tetramer complexes were solvated in a box of TIP3P water with the box boundary extending 20 Å from the nearest peptide atom (resulting in 18,363 water molecules in each system). All Lys, Arg, Glu and Asp residues were in their charged states. His273 was considered ε-protonated. Prior to equilibration, the solvated systems were sequentially subjected to the following steps of modeling:

1. An equilibration of peptides in water for 500 picoseconds (ps) with peptides fixed (Belly dynamics)
2. Energy minimization of the entire system for 5,000 steps
3. Additional equilibration at constant temperature (200 K) and constant pressure (1 atm) dynamics for ~1 nanosecond (ns) with tetramer configuration fixed to assure a reasonable starting density around 1 g/cc
4. Additional energy minimization of the entire system for 5,000 steps
5. Stepwise heating at constant volume to bring the temperature up to 300 K in 3 ns
6. Constant volume simulations for 10 ns with a constraint force constant of 10 kcal/mol applied only on backbone heavy atoms
7. After releasing all constraining forces within the next 20 ns of the equilibration, full molecular dynamics simulations at constant temperature (Langevin thermostat) and constant volume were performed for 1 microsecond (ms) for both the wild-type and mutant YG-box tetramer complexes

All trajectories were calculated using the PMEMD module of the Amber2020 software with a 2 femtosecond (fs) time step. Long range coulombic interactions were handled using the particle mesh Ewald (PME) method with a 9 Å cut-off for the direct interactions. The amino-acid parameters were selected from the FF14SB forcefield of Amber 2020.

**References**:

1. *AMBER 2020*. University of California; 2020.

2. Webb B, Sali A. Protein Structure Modeling with MODELLER. *Methods Mol Biol*. 2021;2199:239-255. doi:10.1007/978-1-0716-0892-0_14

3. Gupta K, Wen Y, Ninan NS, et al. Assembly of higher-order SMN oligomers is essential for metazoan viability and requires an exposed structural motif present in the YG zipper dimer. *Nucleic Acids Research*. 2021;49(13):7644-7664. doi:10.1093/nar/gkab508
